# 

**Published:** 1894-05

**Authors:** 


### CONTENTS.

ORIGINAL COMMUNICATIONS—

The Duty of the Physician to His Patient. By W. H. Elliott, M.D., Savannah, Ga. .... 129

Operation for Fibro-Cystic Sarcoma Involving the Right Inferior Maxillary Bone. By J. McFadden Gaston, M.D., Atlanta, Ga. .... 142

A Plea for the Obstetric Binder. By Joseph Eve Allen, M.D., Augusta, Ga. .... 151

Granular Endometritis.—Menorrhagia from Fungous Endometritis.—Dysmenorrhea Due to Anteflexion. By Augustin H. Goelet, M.D., New York .... 156

SOCIETY REPORTS—

Georgia State Medical Association .... 160

EDITORIAL—

The Medical Association .... 170

Some Misdirected Letters .... 172

SELECTIONS AND ABSTRACTS—

Obstetrics and Gynecology .... 174

General Medicine and Therapeutics .... 179

Surgery .... 185

MEDICAL ITEMS .... 190

BOOK REVIEWS .... 192

ADVERTISERS' INDEX TO ADVERTISEMENTS .... xiv

CLASSIFIED INDEX TO ADVERTISEMENTS .... xxxvii

ITEMS OF INTEREST .... x, xi, xii, xiii

